# Supplementary material for: Swift and extensive Omicron outbreak in China after sudden exit from ‘zero-COVID’ policy
Source: Nat Commun. 2023 Jul 1;14:3888. doi: 10.1038/s41467-023-39638-4 (PMC10314942; doi:10.1038/s41467-023-39638-4)
Supplement: Supplementary file 1 — Supplementary Information [file 41467_2023_39638_MOESM1_ESM.pdf]

Swift and extensive Omicron outbreak in China after sudden exit from ‘zero-COVID’ policy

Emma E. Goldberg[1], Qianying Lin[1], Ethan O. Romero-Severson[1], Ruian Ke[1,*]

[1]Theoretical Biology and Biophysics (T-6), Los Alamos National Laboratory, Los Alamos, NM 87545, USA

*Corresponding author email: rke@lanl.gov

Supplementary Material

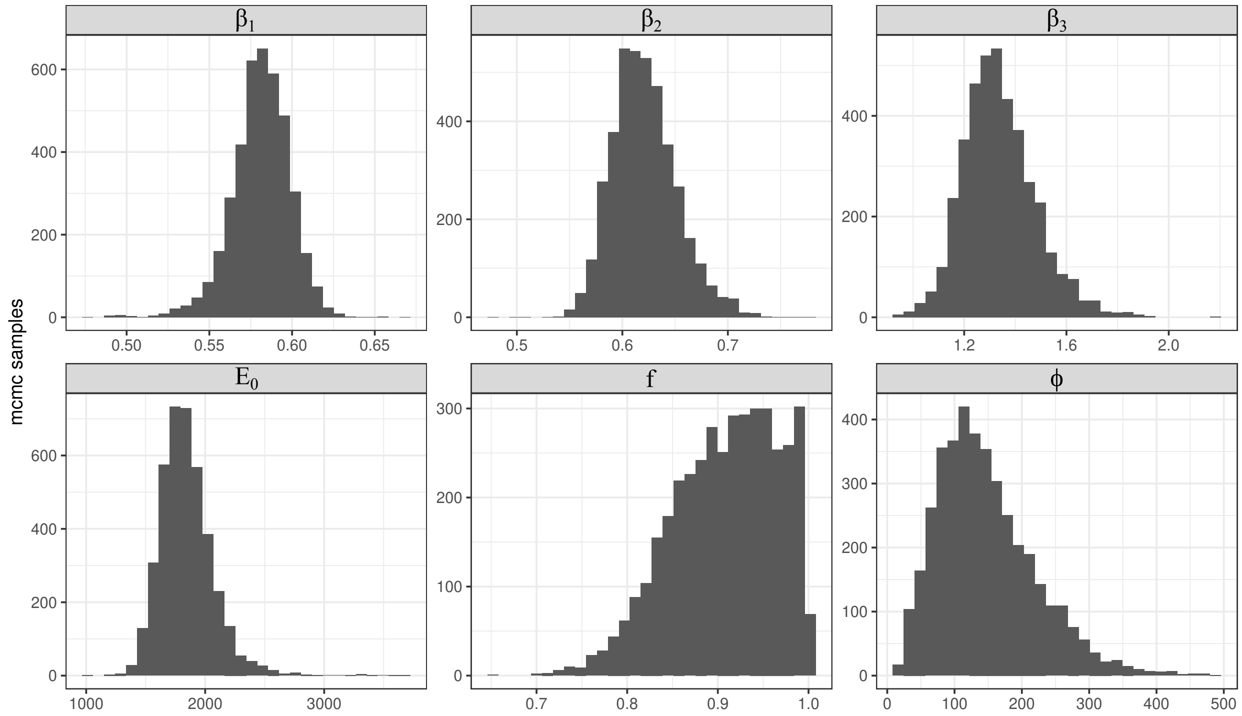

Figure S1: **Posterior distributions of model parameters.** The free parameters in our model were the transmission rate for each time period (β_i), an initial condition on the number of exposed people on Oct. 22 (E_0), the proportion of infected people who eventually develop symptoms (f), and an overdispersion parameter for the count data (ϕ).

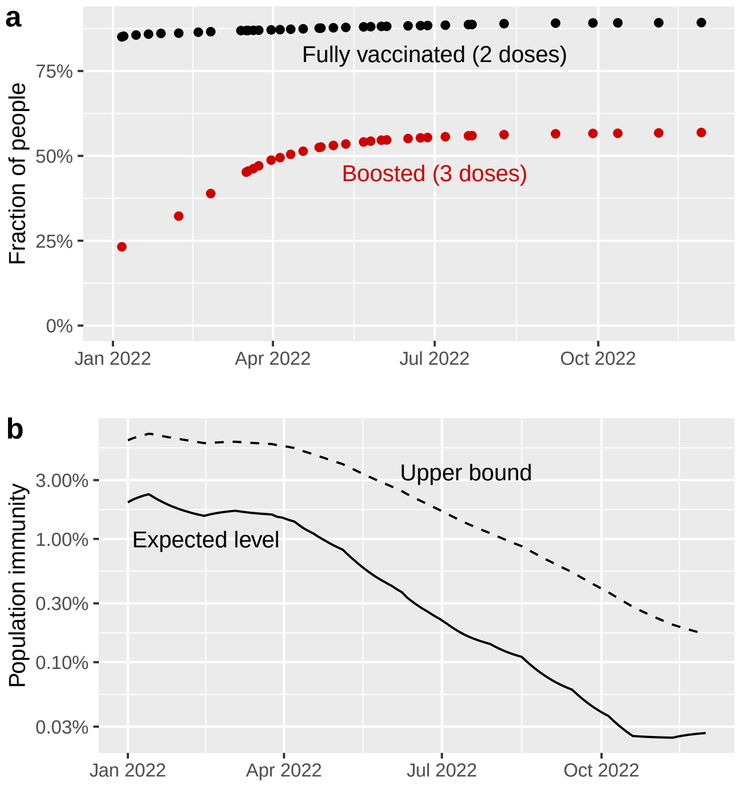

Figure S2: **Population immunity induced by vaccination in China was very low against Omicron infection.** Most individuals received their second or third dose of vaccine prior to 2022 or Apr. 2022, respectively. Combined with relatively low vaccine effectiveness, this led to overall low population immunity, despite efforts to increase vaccination coverage in late 2022. **(a)** Fraction of people vaccinated according to the number of doses received, as reported in Ref. [14]. Black and red dots represent fully vaccinated and boosted individuals, respectively. **(b)** Estimated levels of population immunity against Omicron infection in China using data in (a) and the vaccine effectiveness function in Ref. [15]. Expected values and upper bound values were shown as solid and dashed lines, respectively. Note that the vertical axis is plotted on a log scale.

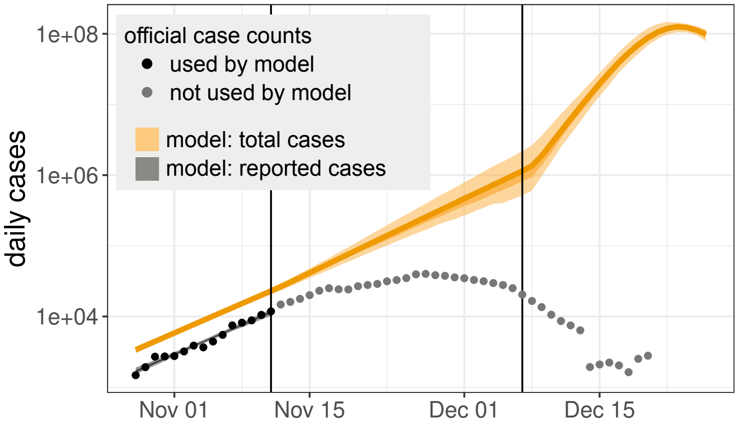

Figure S3: **Sensitivity of inferred epidemiological dynamics to under-reporting of official case counts.** If the official case counts up to Nov. 11 included only half of the true cases ($w = 0.5$), a good quality model fit is still obtained and the epidemic trajectory is similar. Orange bands show the 50% and 95% CrI for the model-predicted total number of cases, and gray bands show the same CrI for the official case counts under this assumption of under-reporting. Other figure components are as in Fig. 1a.

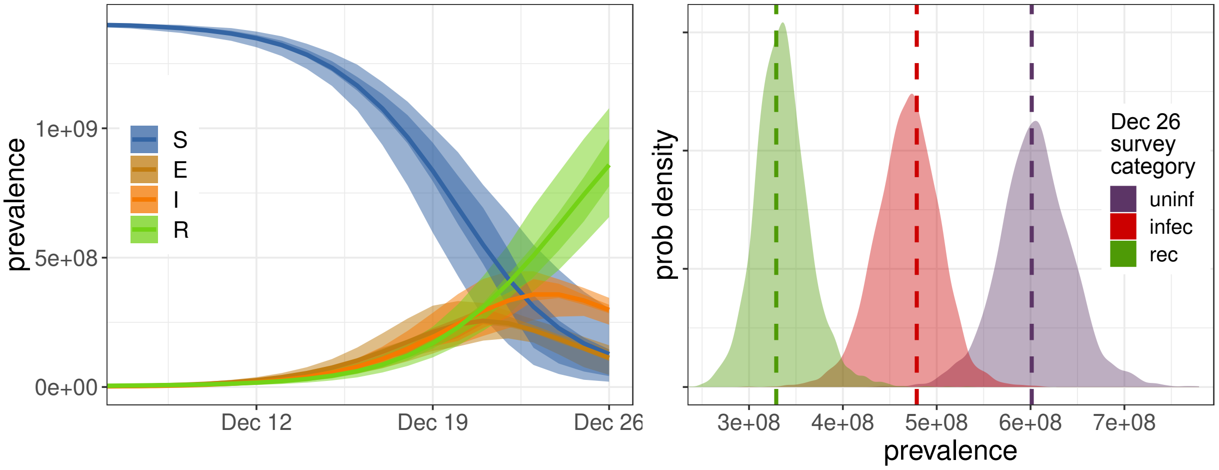

Figure S4: **Sensitivity of inferred epidemiological dynamics to perturbation of the Dec. 26 survey data.** Even after moving 20% of survey responses out of the infected and recovered categories, and into the uninfected category (right), the pace and magnitude of the epidemic are little changed (left). Figure components are as in Fig. 1bd, showing the model-predicted SEIR dynamics with credibility intervals (left) and the model-predicted number of people in each survey category (colored and shaded areas) compared with the data (vertical lines).

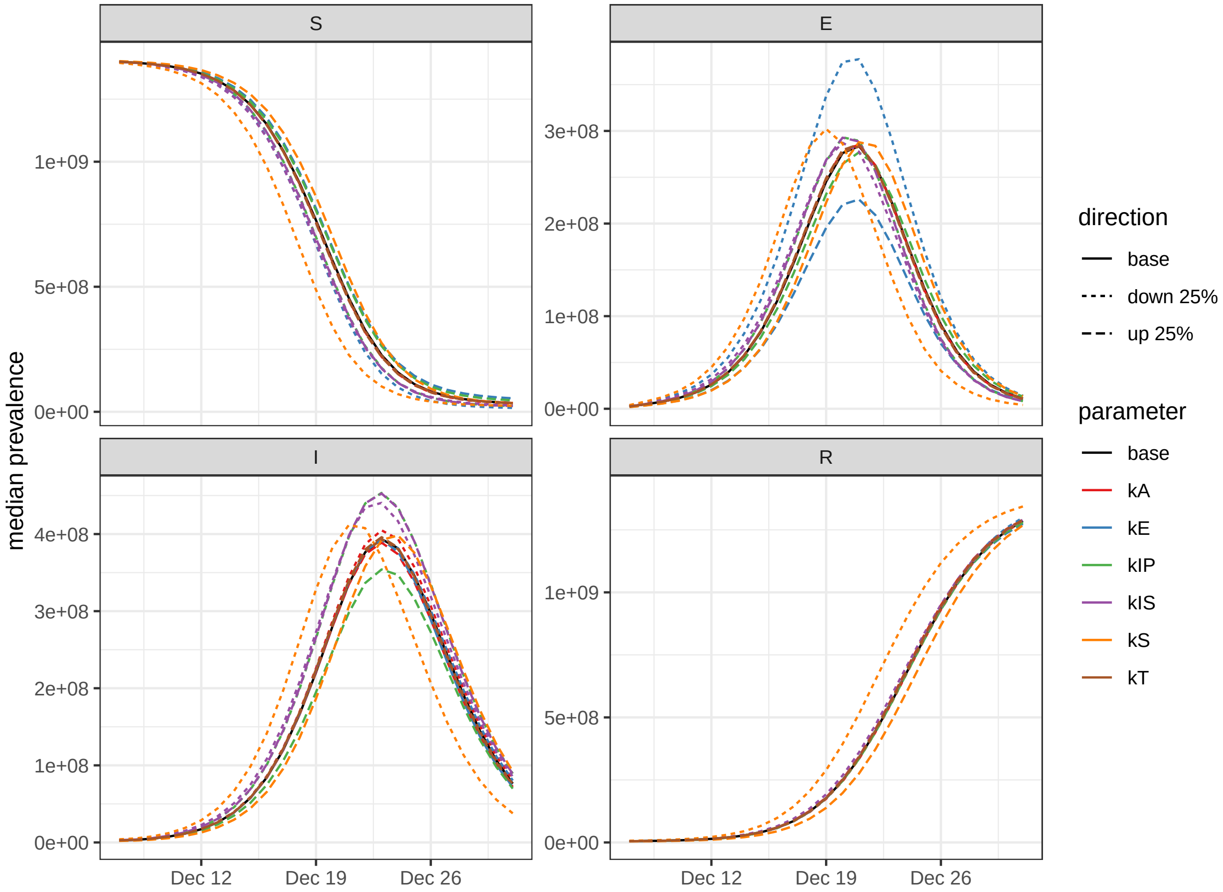

Figure S5: **Sensitivity of inferred epidemiological dynamics to perturbations of the parameter values.** The four panels show the changes in the four epidemiological states (S, E, I and R, as indicated in the titles of the panels). Model parameters (colored lines) are defined in Fig. 2 and Eq. 1, and baseline parameter values are stated in Table S3. Dotted and dashed lines show the model results when a parameter value was decreased or increased by 25%, respectively.

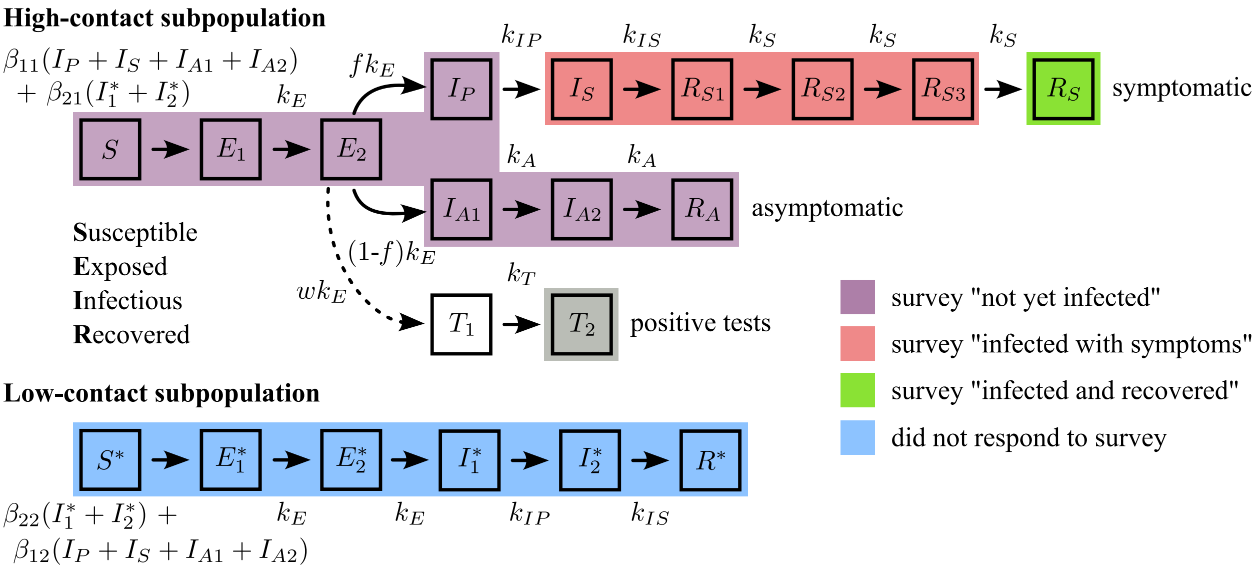

Figure S6: **Schematic of the SEIR-type model with population sub-structure.** This model extends the SEIR-type model in the main text (Fig. 2) by adding a subpopulation that has low contact rates. We assume that both the official case count data and the Dec. 26 survey data were obtained only from the subpopulation with high contact rates. The transmission parameters for contacts within each subpopulation are β_{11} and β_{22}, with $\beta_{22} = \beta_{11}/2$. The transmission parameter for contacts between individuals from different subpopulations is $\beta_{12} = \beta_{21} = \beta_{11}/10$ or $\beta_{11}/100$. Note that we still allow different transmission rates for different time periods (Fig. 1ac), so the overall amount of contact within and between subpopulations increases as control policies are relaxed. The model structure for the low-contact subpopulation is simpler because there is no need to distinguish people who are asymptomatic or recovered but still symptomatic, since we assume that people in this subpopulation did not respond to the survey.

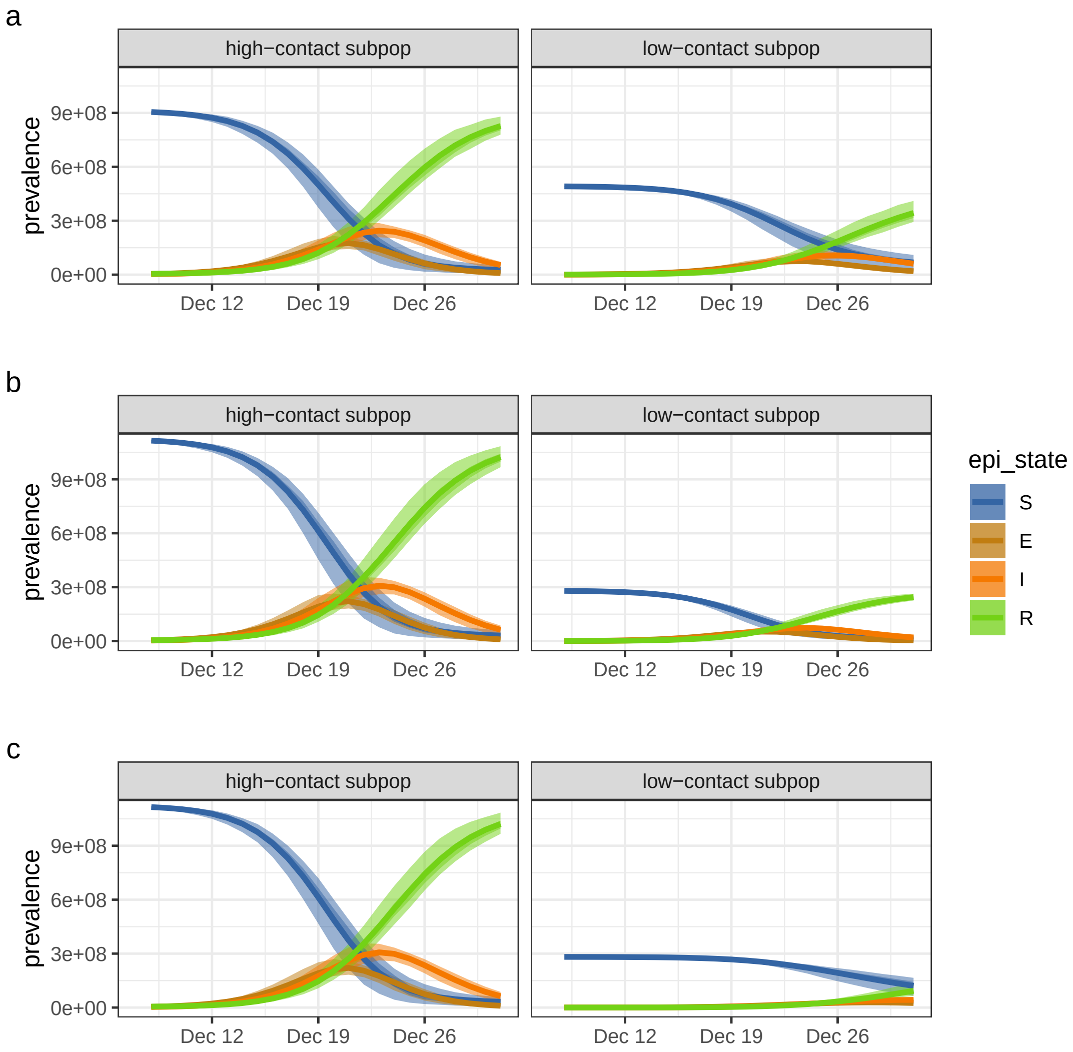

Figure S7: **Epidemic dynamics under different assumptions about population sub-structure.** (a) The low-contact group is 35% of the entire population. (b) The low-contact group is 20% of the entire population. (c) The transmission rate between the high- and low-contact subpopulations is 1% of that within the high-contact subpopulation, in contrast with 10% for (a) and (b). Figure components are as in Fig. 1d, showing the model-predicted SEIR dynamics with credibility intervals.

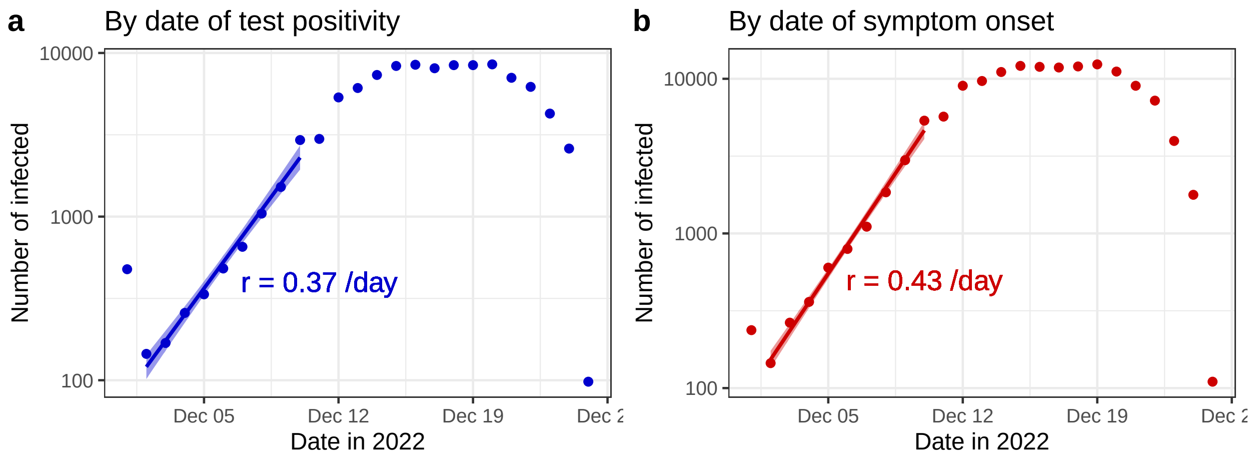

Figure S8: **Number of infected individuals (dots) by date of test positivity (a) or by date of symptom onset (b) in the Sichuan survey data, and the exponential growth rates, *r*, estimated from regression analyses (lines).** Shaded areas along the regression lines show the 95% confidence interval for the regression. The exponential growth rates, *r*, were estimated using data between Dec. 2 and 10 (9 data points for each regression), during which the number of infected people grew exponentially. Data on Dec. 1 was not used in estimation because the numbers on the day likely include individuals who tested positive or had symptom onset before Dec. 1.

period	increase parameter	median [95% CrI]
Oct 28 – Nov 11	β	0.582 [0.546, 0.615]
	r	0.148 [0.129, 0.164]
	R	1.57 [1.49, 1.64]
Nov 11 – Dec 7	β	0.618 [0.563, 0.681]
	r	0.165 [0.138, 0.195]
	R	1.65 [1.53, 1.79]
Dec 7 – Dec 26	β	1.32 [1.09, 1.65]
	r	0.421 [0.351, 0.508]
	R	3.13 [2.66, 3.79]

Table S1: **Estimated values for the transmission rate (β), intrinsic rate of increase (r) and reproductive number (R) during each modeled time period.** Note that the values of r and R reported for the period between Dec. 7 and Dec. 26 represent the growth rate and the reproductive number during the exponential growth of the outbreak.

**Q: Have you been infected with COVID-19?<br>(If no test conducted, please reply based on your judgement.)**		
Options	Votes	Percentage
Not yet infected.	12,671	26%
Infected without symptoms.	1264	2%
Infected with symptoms.	19,345	40%
Infected and recovered.	14,617	30%
**Q: For symptomatic infections, how severe was it?**		
Options		Percentage
Severe.		35%
Relatively severe.		41.1%
I felt okay.		15.9%
Relatively mild.		6.9%
Mild.		1%
**Q: For recovered patients, how long did the symptoms last?**		
Options		Percentage
1-2 days		10%
3-4 days		22.5%
5-6 days		28.2%
7-8 days		26.8%
9-10 days		8.1%
11+ days		4.4%

Table S2: **Questions, options, and response results in the Dec. 26 nation-wide survey, reported by RenSheTong [13].**

Parameter	Description	Formula	Mean value	Std err	Reference
p_{inc}	incubation period	$2/k_E + 1/k_{IP}$	3.4 days	0.3 day	[37]
p_{pre}	pre-symptomatic period	$1/k_{IP}$	1.5 days	0.2 day	approx. from [38]
p_{sym}	symptomatic period	$1/k_{IS} + 3/k_S$	5.7 days	0.4 day	est. from [13]
p_{gen}	generation interval	$2/k_E + (1/k_{IP} + 1/k_{IS})/2$	3.4 days	0.7 day	approx. from [16]
p_{tst}	time to PCR test result	$1/k_T$	2 days	0.5 day	
k_{IP}	pre-sympt to sympt	$1/p_{pre}$	1/1.5 /day		
k_E	still exposed	$2/(p_{inc} - 1/k_{IP})$	2/1.9 /day		
k_{IS}	sympt to recovered	$1/(2[p_{gen} - 2/k_E] - 1/k_{IP})$	1/1.5 /day		
k_S	recovering	$3/(p_{sym} - 1/k_{IS})$	3/4.2 /day		
k_A	asymptomatic	$2/(1/k_{IP} + 1/k_{IS})$	1/1.5 /day		
k_T	testing	$1/p_{tst}$	1/2 /day		

Table S3: **Parameter values and distributions used in the model.** The rate parameters, k, are defined in Fig. 2, and the values here were used in the main model fit. To account for uncertainty in those values, we additionally drew from normal distributions for periods shown here, p, and used the consequent values of the k parameters; all values were constrained to be positive. In addition to this table of fixed parameters, the free parameters of the model are shown in Fig. S1.
